# Urine color as an indicator of urine concentration in pregnant and lactating women

**DOI:** 10.1007/s00394-015-1085-9

**Published:** 2015-11-16

**Authors:** Amy L. McKenzie, Colleen X. Muñoz, Lindsay A. Ellis, Erica T. Perrier, Isabelle Guelinckx, Alexis Klein, Stavros A. Kavouras, Lawrence E. Armstrong

**Affiliations:** 1 Human Performance Laboratory, Department of Kinesiology, University of Connecticut, 2095 Hillside Road, U1110, Storrs, CT 06269-1110 USA; 2 Department of Health Sciences and Nursing, University of Hartford, 200 Bloomfield Ave, West Hartford, CT 06117 USA; 3 Centre for Heart Lung and Vascular Health, University of British Columbia, 1147 Research Road, Kelowna, BC V1V 1V7 Canada; 4 Danone Research, RD 128, 91767 Palaiseau, France; 5 Human Performance Laboratory, Department of Health, Human Performance and Recreation, University of Arkansas, 155 Stadium Drive, HPER 321, Fayetteville, AR 72701 USA

**Keywords:** Fluid intake, Hydration status, Biomarker, Urine color, Pregnant women, Lactating women

## Abstract

**Aim:**

Urine concentration measured via osmolality (*U*_OSM_) and specific gravity (*U*_SG_) reflects the adequacy of daily fluid intake, which has important relationships to health in pregnant (PREG) and lactating (LACT) women. Urine color (*U*_COL_) may be a practical, surrogate marker for whole-body hydration status.

**Purpose:**

To determine whether *U*_COL_ was a valid measure of urine concentration in PREG and LACT, and pair-matched non-pregnant, non-lactating control women (CON).

**Methods:**

Eighteen PREG/LACT (age 31 ± 1 years, pre-pregnancy BMI 24.3 ± 5.9 kg m^−2^) and eighteen CON (age 29 ± 4 years, BMI 24.1 ± 3.7 kg m^−2^) collected 24-h and single-urine samples on specified daily voids at five time points (15 ± 2, 26 ± 1, and 37 ± 1 weeks gestation, 3 ± 1 and 9 ± 1 weeks postpartum during lactation; CON visits were separated by similar time intervals) for measurement of 24-h *U*_OSM_, *U*_SG_, and *U*_COL_ and single-sample *U*_OSM_ and *U*_COL_.

**Results:**

Twenty-four-hour *U*_COL_ was significantly correlated with 24-h *U*_OSM_ (*r* = 0.6085–0.8390, *P* < 0.0001) and 24-h *U*_SG_ (*r* = 0.6213–0.8985, *P* < 0.0001) in all groups. A 24-h *U*_COL_ ≥ 4 (AUC = 0.6848–0.9513, *P* < 0.05) and single-sample *U*_COL_ ≥ 4 (AUC = 0.9094–0.9216, *P* < 0.0001) indicated 24-h *U*_OSM_ ≥ 500 mOsm kg^−1^ (representing inadequate fluid intake) in PREG, LACT, and CON.

**Conclusions:**

Urine color was a valid marker of urine concentration in all groups. Thus, PREG, LACT, and CON can utilize *U*_COL_ to monitor their daily fluid balance. Women who present with a *U*_COL_ ≥ 4 likely have a *U*_OSM_ ≥ 500 mOsm kg^−1^ and should increase fluid consumption to improve overall hydration status.

## Introduction

Water is an essential nutrient [1] and plays a vital role in metabolism by maintaining cellular shape, supporting cellular functions, and serving as a transport medium for nutrients and wastes [2]. Water is especially important to women who are pregnant or nursing. During gestation, plasma volume expands [3], and amniotic fluid protects the developing child [4]. An average woman gains about 11 kg of body mass during pregnancy, and a large portion of this gain (approximately 7–8 L) is due to water retention [5]. During the postpartum period, nursing mothers experience an increased water loss via breast milk, representing approximately 700 ml per day at 8 weeks postpartum [6, 7]. These increased physiological needs for water pose a challenge to the neuroendocrine mechanisms that regulate fluid–electrolyte balance and suggest evolving water requirements from conception to lactation.

Despite the importance of water to both the mother and developing child, research on the regulation of total body water balance, daily water needs, and biomarkers of hydration in pregnant and lactating women is scarce. Even less is known about how maternal hydration may impact either mother or fetus during pregnancy or into early life. Consequently, the dietary reference intakes of water during pregnancy in both Europe and the USA (2.3 and 3.0 L day^−1^, respectively) are based only upon the increased amount of water needed to offset the increased caloric intake during pregnancy (i.e., 300 ml day^−1^ for 300 kcal day^−1^) [8, 9]. During lactation, adequate intake of total water increases to 2.7 [8] and 3.8 [9] L day^−1^ in Europe and the USA, respectively, to offset water lost in breast milk.

While dietary reference intakes provide guidelines for adequate intake for the population [8, 9], individual water needs vary greatly depending upon personal water intake via the diet, and personal water losses through physical activity, environmental, and other factors. Among the various sources of daily water loss, urine output has been identified as one way to assess hydration in the individual. Urine volume and concentration represent the end result of intake and loss, after accounting for differences in sweat loss and dietary solute load. Manz et al. [10] suggested that 24-h urine concentration reflects the kidney’s self-regulation of fluid volume deficit or excess, and provides an indication of the sum of all water gains and losses in healthy individuals. Other recent publications [11, 12] support this concept with data showing that, in healthy men and non-pregnant women, urinary hydration biomarkers including urine osmolality (*U*_OSM_) and specific gravity (*U*_SG_) represent inter- and intra-individual differences of daily fluid intake in high- versus low-volume consumers. While useful in a clinical or laboratory setting, *U*_OSM_ is not readily available to the general population. However, urine color (*U*_COL_), a tool that requires little cost, laboratory instrumentation, and expertise, may be valuable as a surrogate hydration marker for daily monitoring of urine concentration. While *U*_COL_ has been demonstrated to track changes in daily hydration habits in the general adult population [13–15], no study has assessed its utility or validity in pregnant or lactating women. During pregnancy, serum osmolality is reduced approximately 8–10 mOsm kg^−1^, changing the point of isotonicity between urine and blood at the kidney, potentially impacting free water clearance and *U*_OSM_ and thus potentially interrupting the previously reported relationship between *U*_OSM_ and *U*_COL_ in non-pregnant women. Additionally, some research reports increased void frequency [16] and increased 24-h urine volume with lower 24-h *U*_OSM_ in pregnant women [17], but decreased void frequency [16] and decreased 24-h urine volume with higher 24-h *U*_OSM_ in lactating women [18]. Given that increased 24-h urine volume is associated with lighter urine color and lower osmolality in non-pregnant, non-lactating women [14], this suggests the relationship between *U*_COL_ and *U*_OSM_ may be skewed in, or between, pregnant and lactating women compared to control women and warrants further investigation.

Thus, the purpose of this investigation was to determine whether *U*_COL_ was a valid measure of urine concentration in pregnant and lactating women, as well as pair-matched non-pregnant and non-lactating controls. The validity of urine color was evaluated in two ways: first, by determining the relationships between 24-h *U*_COL_ and biomarkers of urine concentration, specifically 24-h *U*_OSM_ and 24-h *U*_SG_, utilizing a Spearman’s rank-order correlation, and second, by determining the diagnostic accuracy of *U*_COL_ (24-h and single samples, separately), to accurately identify *U*_OSM_ ≥ 500 mOsm kg^−1^, utilizing receiver operating characteristic curve analysis, sensitivity, and specificity. Maintaining *U*_OSM_ under this cutoff has been associated with ensuring adequate daily fluid intake and urine output for the reduction in risk for urolithiasis or decline in kidney function, as well as avoidance of high plasma vasopressin concentrations often linked to disease states [19].

## Methods

### Subjects

Twenty pregnant women and 18 non-pregnant, control women of similar age, height, and weight participated in this study. Pregnant women enrolled in the study prior to 16-week gestation and reported to the laboratory five times over approximately 8 months. All pregnant women enrolled in the study were breastfeeding during the two postpartum visits. Two pregnant participants were removed from data analysis: one who withdrew from the study and one who developed a gestational condition affecting fluid balance. Non-pregnant, non-lactating control women reported to the laboratory at intervals similar to the pregnant/lactating women and were taking a combination drug oral contraceptive; samples were collected from control women only during the early follicular phase of their menstrual cycle (placebo phase of their pill pack) to limit any effect of exogenous estrogen on osmotically influenced components of fluid balance [20] and to ensure data collection occurred at the same time during their cycle at each visit. Exclusion criteria included use of tobacco products, participation in exercise >7 h per week, the presence of a health condition (e.g., type 2 diabetes, polycystic ovarian syndrome) or prescription of a medication that would alter fluid balance, or the development of a gestational condition that would alter fluid balance. The University’s Institutional Review Board approved this study, and thus, all study procedures were performed in accordance with ethical standards specified by the Declaration of Helsinki.

### Experimental design

**Fig. 1:**
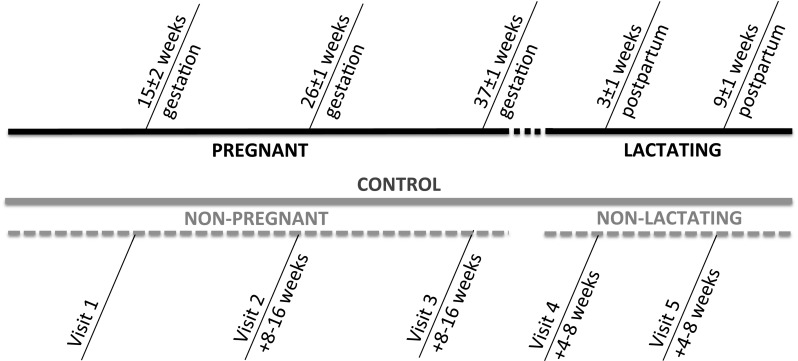
Experimental time line of visits and data collection. Participants collected urine at five time points across approximately 8 months. For pregnant and lactating women, respectively, time indicates their gestational age relative to their last menstrual period and time relative to delivery. For control women, time indicates the time passed since the former visit. Pregnant and lactating women are the same participants, and non-pregnant and non-lactating women are the same participants, but at different time points. These four groups were utilized in correlational analyses with visits 1–3 and visits 4–5 pooled. For diagnostic accuracy analyses, data from non-pregnant and non-lactating women (visits 1–5) were pooled

Data were collected from pregnant participants at the end of the first (15 ± 2 weeks gestation), second (26 ± 1 weeks gestation), and third (37 ± 1 weeks gestation) trimesters and at 3 ± 1 and 9 ± 1 weeks postpartum during lactation. Non-pregnant, non-lactating control women were pair-matched on the basis of age, height, and body mass (compared to pre-pregnancy body mass in pregnant participants) and reported for testing at similar intervals to the pregnant/lactating participants (Fig. 1). Participants self-reported age, parity, and pre-pregnancy body mass. Prior to each visit, women were instructed to eat and drink as they normally would. At each visit, height and body mass were recorded. Height was measured with a stadiometer, and weight was measured to the nearest 0.01 kg (Health O Meter, Model 349KLX, Alsip, IL). Body mass index was calculated as kg m^−2^. Researchers provided each participant with clean urine containers, and the women collected all urine for 24 h before each visit in a large container along with specific single, spot urine samples as follows: participants collected a single sample of their last urine void of the day and consumed 200 ml of water before going to sleep, collected a single sample of any overnight urine voids (thus, the number of overnight urine samples varied between women), and collected a single sample of their first urine void of the day resulting in a minimum of two single, spot samples of urine for each woman. After measurements were taken on the single samples, these single samples were pooled with the remaining collection to form a complete 24-h urine sample; the same measurements were taken on the full 24-h urine sample.

Urine osmolality, specific gravity, and color were measured for each urine sample. Urine osmolality was determined by freezing point depression (Advanced Instruments Inc., Model 3320, Norwood, MI; CV = 0.3 %) in duplicate, and *U*_SG_ was determined by manual refractometry (Reichert Technologies, Model TS-400, Depew NY). Urine color was determined by a single researcher in a well lit room by placing the sample in a clear container and on a white background next to a previously published chart [21]. The researcher recorded the number of the chart color that most closely matched the urine sample; if the color of the urine sample appeared to fall between two colors on the chart, the researcher chose the darker of the two colors for consistency in assessment technique.

### Statistical analysis

Differences between groups in demographic characteristics were assessed using independent samples *t* tests at the end of the first trimester (15 ± 2 weeks gestation, visit 1) to compare pregnant (PREG) to non-pregnant (NP) women. Correlations between 24-h *U*_COL_ and *U*_SG_ and *U*_OSM_ were evaluated in PREG (visits 1–3 pooled), NP (visits 1–3 pooled), lactating women (LACT; visits 4–5 pooled), and non-lactating women (NL; visits 4–5 pooled) with Spearman’s rank-order correlations. For diagnostic accuracy analyses, NP and NL were pooled into one group of control women (CON). The diagnostic accuracy of *U*_COL_ in 24-h and single samples was assessed in PREG, LACT, and CON using receiver operating characteristic (ROC) curve analysis to determine the ideal *U*_COL_ criterion value to identify *U*_OSM_ ≥ 500 mOsm kg^−1^ [8, 19]. Repeated measures in ROC curve analyses were not accounted for to allow a more conservative analysis [22]. Differences in area under the curve (AUC) were evaluated using methods previously described [23, 24] and corrected with a Bonferroni adjustment to account for multiple comparisons. Follow-up calculations of sensitivity and specificity utilizing the criterion value identified by the ROC curve analysis were performed. Level of significance was set a priori at *P* < 0.05.

## Results

### Demographics and correlations

**Fig. 2:**
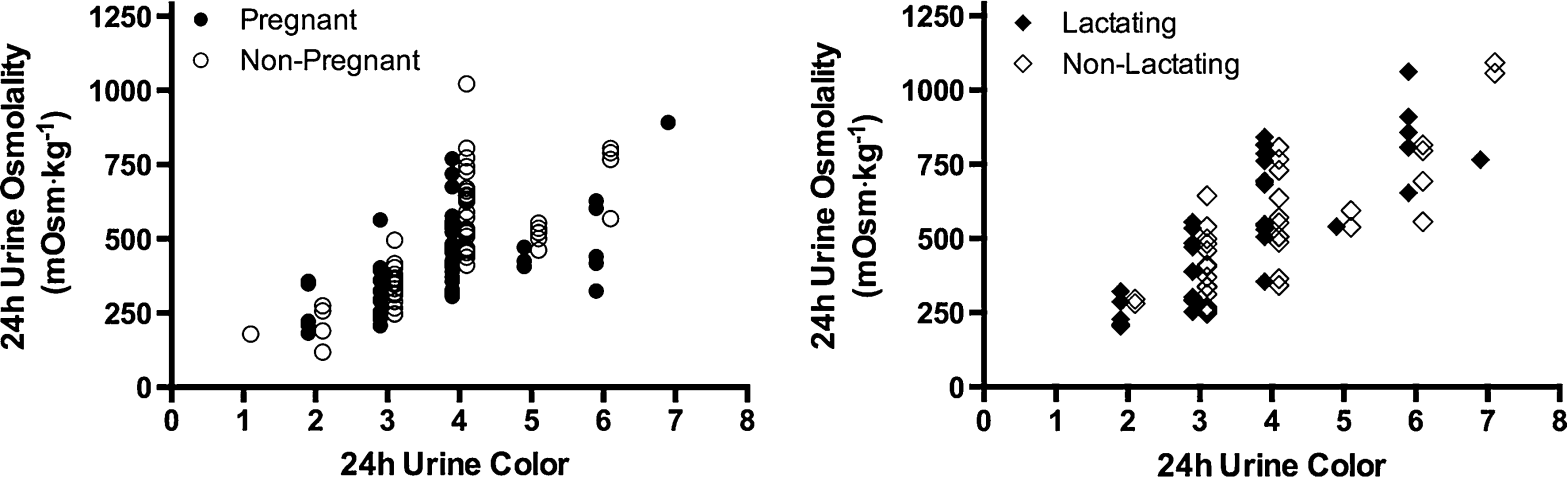
Relationships of 24-h *U*_COL_ to 24-h *U*_OSM_ during pregnancy and lactation. Individual data points represent 24-h *U*_COL_ and 24-h *U*_OSM_ during PREG (visits 1–3 pooled, *r* = 0.6085, *P* < 0.0001), NP (visits 1–3 pooled, *r* = 0.7826, *P* < 0.0001), LACT (visits 4–5 pooled, *r* = 0.8390, *P* < 0.0001), and NL (visits 4–5 pooled, *r* = 0.7736, *P* < 0.0001)

**Fig. 3:**
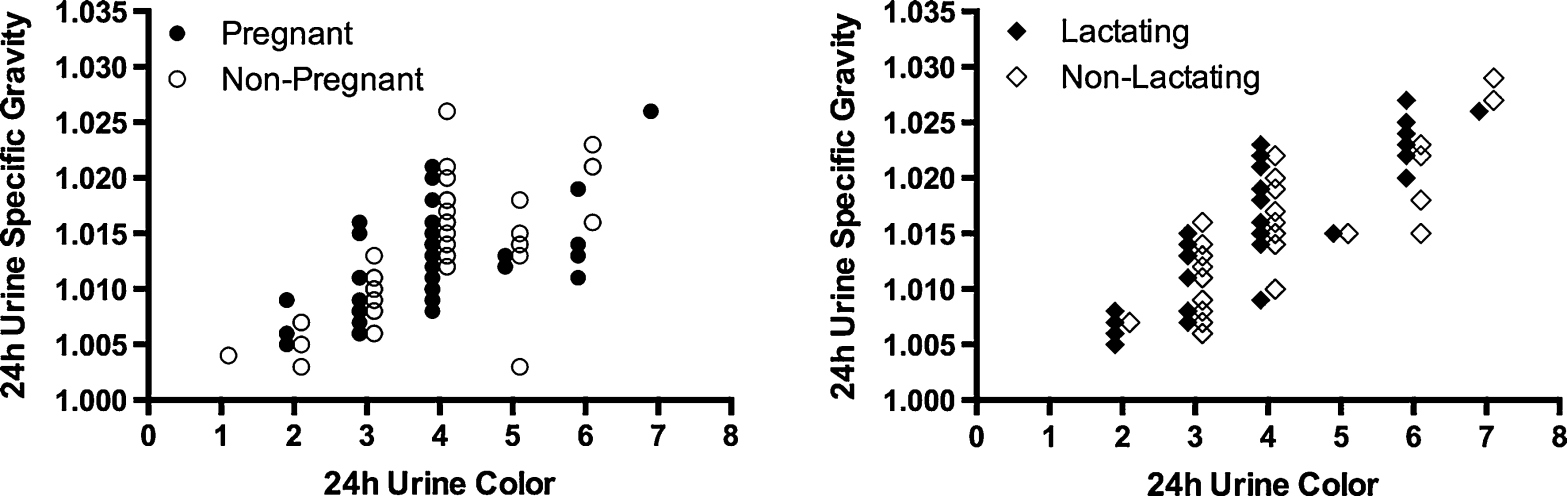
Relationships of 24-h *U*_COL_ to 24-h *U*_SG_ during pregnancy and lactation. Individual data points represent 24-h *U*_COL_ and 24-h *U*_SG_ during PREG (visits 1–3 pooled, *r* = 0.6213, *P* < 0.0001), NP (visits 1–3 pooled, *r* = 0.7416, *P* < 0.0001), LACT (visits 1–3 pooled, *r* = 0.8985, *P* < 0.0001), and NL (visits 1–3 pooled, *r* = 0.8039, *P* < 0.0001)

At the end of the first trimester, pregnant women were similar to their matched controls in age, height, body mass, self-reported pre-pregnancy body mass, and pre-pregnancy BMI (Table 1; *P* > 0.05). Twenty-four-hour *U*_COL_ was significantly correlated with 24-h *U*_OSM_ (*r* = 0.61–0.84, *P* < 0.0001; Fig. 2) and 24-h *U*_SG_ (*r* = 0.62–0.90, *P* < 0.0001; Fig. 3) in all groups.

**Table 1:** Participant demographics and anthropometrics

	Pregnant/lactating women	Control women	*t*	*P*
*n*	18	18		
Parity (%)				
Nulliparous	28	89		
Primiparous	55	11		
Multiparous	17	0		
Age (years)	31 ± 3	29 ± 4	1.382	0.176
Height (m)	1.66 ± 0.07	1.64 ± 0.08	0.752	0.458
Body mass (kg)	69.58 ± 18.57	64.83 ± 13.84	0.870	0.390
Self-reported pre-pregnancy body mass (kg)	66.89 ± 19.24	64.70 ± 14.28	0.368	0.715
Pre-pregnancy BMI (kg m^−2^)	24.3 ± 5.9	24.1 ± 3.7	0.134	0.895

### Diagnostic accuracy of 24-h urine color

**Fig. 4:**
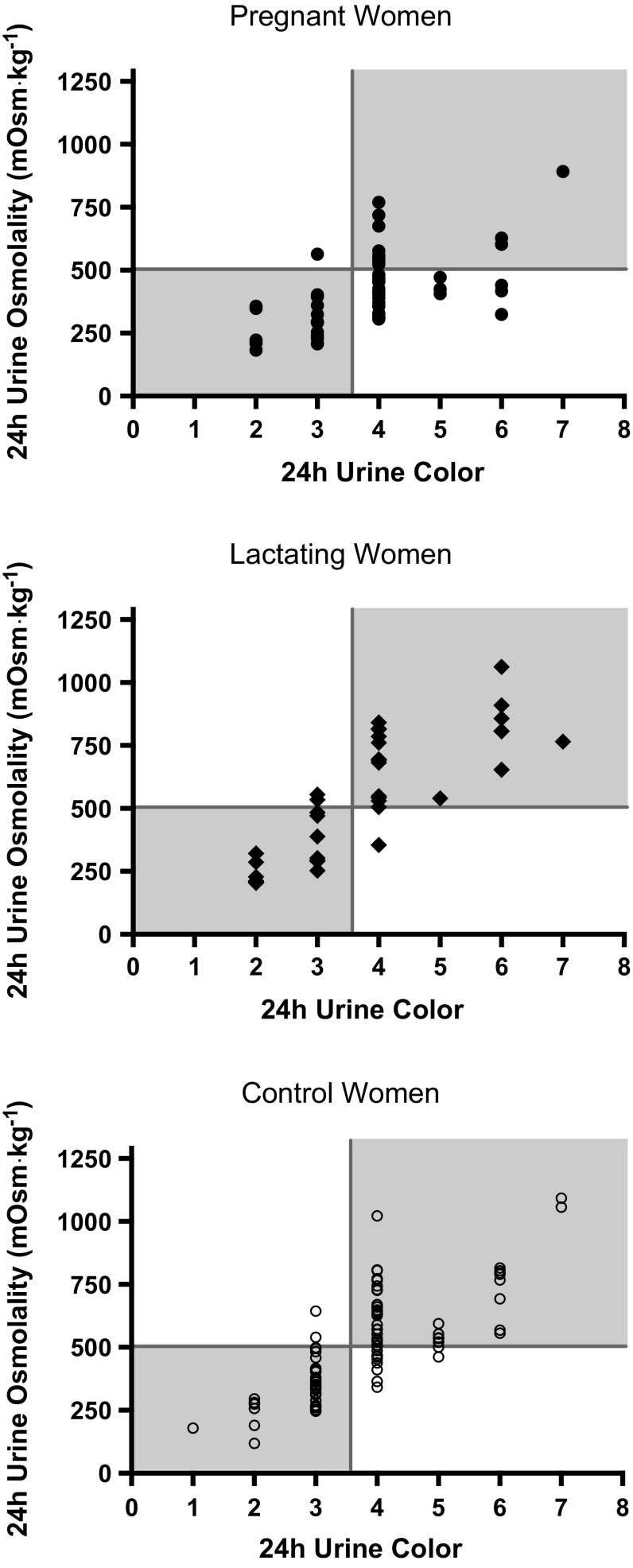
Contingency plots for 24-h *U*_COL_ and 24-h *U*_OSM_. *Vertical line* represents the *U*_COL_ ≥ 4 criterion value determined by the ROC curve analysis to identify *U*_OSM_ ≥ 500 mOsm kg^−1^ (*horizontal line*)

Receiver operating characteristic curve analysis revealed that 24-h *U*_COL_ was a useful diagnostic tool to identify 24-h *U*_OSM_ ≥ 500 mOsm kg^−1^ in PREG, LACT, and CON (AUC = 0.685–0.951, *P* < 0.05; Table 2). Area under the curve was significantly different between PREG and LACT (*P* = 0.002) as well as PREG and CON (*P* = 0.007). However, despite significant differences in AUC, all AUC were statistically significant indicating that 24-h *U*_COL_ is an accurate indicator of 24-h *U*_OSM_ ≥ 500 mOsm kg^−1^ for all groups, and ROC curve analyses identified the same criterion value with the highest sensitivity and specificity in all groups. A 24-h *U*_COL_ criterion value of 4 or higher correctly identified 24-h *U*_OSM_ ≥ 500 mOsm kg^−1^ in more than 90 % of the cases in PREG, LACT, and CON (Table 2; Fig. 4); 24-h *U*_COL_ ≥ 4 and ≥5 demonstrated a trade off between higher sensitivity and higher specificity, respectively, in all groups.

**Table 2:** Receiver operating characteristic curve analysis of *U*_COL_ to identify *U*_OSM_ ≥ 500 mOsm kg^−1^

Group	AUC	SE	*P*	Criterion value	Sensitivity (%)	Specificity (%)
24-h *U*_COL_						
Pregnant women	0.6848	0.0774	0.0463	4	92	41
Lactating women	0.9513	0.0355	<0.0001	4	91	93
Control women	0.9109	0.0310	<0.0001	4	96	80
Single-sample *U*_COL_						
Pregnant women	0.9193	0.0190	<0.0001	4	98	70
Lactating women	0.9216	0.0244	<0.0001	4	90	78
Control women	0.9094	0.0205	<0.0001	4	86	88

### Diagnostic accuracy of urine color in single samples

**Fig. 5:**
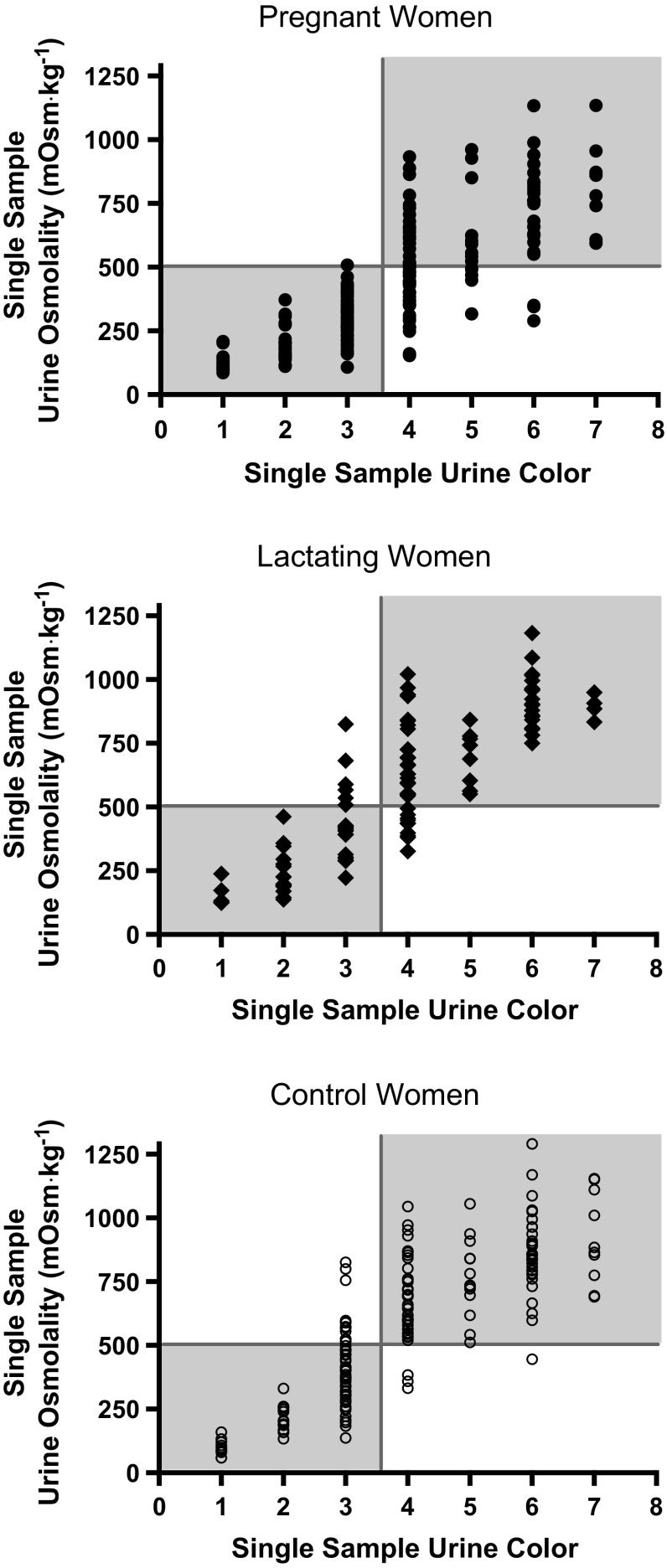
Contingency plots for *U*_COL_ and *U*_OSM_ in single samples. *Vertical line* represents the *U*_COL_ ≥ 4 criterion value determined by the ROC curve analysis to identify *U*_OSM_ ≥ 500 mOsm kg^−1^ (*horizontal line*)

Urine color of single samples also was a useful diagnostic tool in PREG, LACT, and CON to identify *U*_OSM_ ≥ 500 mOsm kg^−1^ based on ROC curve analysis (AUC = 0.909–0.922, *P* < 0.0001; Table 2). Area under the curve was not different between any of the groups (*P* > 0.05). Similar to the 24-h sample ROC curve analysis, single-sample *U*_COL_ criterion values of ≥4 and ≥5 were identified in all groups and represented a trade off between higher sensitivity (*U*_COL_ ≥ 4) and higher specificity (*U*_COL_ ≥ 5). A single-sample *U*_COL_ criterion value of 4 or higher correctly identified *U*_OSM_ ≥ 500 mOsm kg^−1^ in ≥86 % of the cases in all groups (Table 2; Fig. 5).

## Discussion

There is no clear consensus on what represents an optimal 24-h urine concentration, either for the general population or specifically for pregnant and lactating women. Approximately 800 mOsm kg^−1^ has been proposed and used as an upper limit for euhydration [25, 26]. However, 500 mOsm kg^−1^ has been suggested as a threshold for “appropriate” [27] or “optimal” [19] hydration, supported by observed associations between low 24-h fluid intake, low 24-h urine volume, and/or high 24-h urine concentration and the development of kidney stones [28], urinary tract infection [29], hyperglycemia [30], chronic kidney disease [31], and rate of decline in estimated glomerular filtration rate [32, 33]. Given the heightened importance of water balance in pregnancy, it appears prudent to leave a reasonable margin between a target 24-h urine osmolality and the upper limit for euhydration, and thus, 500 mOsm kg^−1^ was deemed appropriate. This value is also supported as a desirable 24-h urine osmolality in the EFSA opinion on dietary reference values for water [8].

We adopted a *U*_OSM_ under 500 mOsm kg^−1^ as a goal for pregnant and lactating women to optimize body water balance [8] and avoid potential negative health consequences [19]. However, the technical requirements of measuring *U*_OSM_ (i.e., instrumentation, expertise) preclude its use during daily activities and emphasize the need for simple, noninvasive hydration assessment techniques such as *U*_COL_ [34–36]. Figures 2 and 3 demonstrate that 24-h *U*_COL_ was significantly correlated with *U*_SG_ and *U*_OSM_ in pregnant and lactating women. While a range of *U*_SG_ and *U*_OSM_ is associated with each *U*_COL_ for pregnant and lactating women, the degree of variation is similar to relationships demonstrated in other subject populations, including non-pregnant women [14], men [37], the elderly [38, 39], and children [40] where correlation coefficients have ranged from 0.48 to 0.93. This variation may be explained by the fact that urine color and urine osmolality are generated by two different mechanisms. Urine color is produced by the concentration of urochome in the urine, which is generated by metabolic processes and independent of diet [41, 42]; urine osmolality is determined by the concentration of solute (e.g., Na^+^, K^+^, Cl^−^, urea) in the urine [43]. Despite variation between measures of urine concentration and urine pigmentation, the two remain significantly correlated and adequate means of assessing hydration status in multiple populations.

Further, ROC curve analyses (Table 2) indicated that *U*_COL_ provided adequate diagnostic accuracy to allow women to successfully monitor *U*_COL_ in order to maintain a urine concentration below 500 mOsm kg^−1^. Because a *U*_COL_ ≥ 4 was associated with high sensitivity (i.e., a high true positive rate), a negative test (i.e., a *U*_COL_ of 1, 2, or 3) largely ruled-out the possibility of *U*_OSM_ being greater than 500 mOsm kg^−1^. Thus, when a *U*_COL_ ≥ 4 is observed, *U*_OSM_ is likely ≥500 mOsm kg^−1^ and warrants increased fluid consumption (assuming no change in solute load) to reduce urine concentration below 500 mOsm kg^−1^. A visual seemingly equal split of single-sample *U*_OSM_ above and below 500 mOsm kg^−1^ at a *U*_COL_ of 4 in pregnant women is present (Figs. 4, 5); this is statistically evident in the trade off between sensitivity and specificity at that criterion value (Table 2). In order to capture as many true positives as possible, the criterion value will favor a higher sensitivity than specificity; thus, a *U*_COL_ of 4 emerges as the criterion value to identify *U*_OSM_ ≥ 500 mOsm kg^−1^ with the greatest sensitivity. A lesser AUC in ROC curve analyses for 24-h urine samples in pregnant women statistically represents this variability (Fig. 4; Table 2). While the AUC for 24-h *U*_COL_ in pregnant women was significantly different from the other groups, the AUC was still statistically significant, and the criterion value was the same as the criterion value identified in the other groups. This implies that the clinical use of 24-h *U*_COL_ in pregnant women to determine whether 24-h *U*_OSM_ is ≥500 mOsm kg^−1^ is still valid, but less diagnostically accurate compared to other groups. Future research should evaluate potential sources of this variation.

Monitoring and optimizing water intake during pregnancy and lactation is important, given the inherent physiological challenges to water balance. Increased daily water intake supports expanded total body water, tissue development during gestation, and water loss due to emesis, if experienced. Increased water intake also offsets the water secreted in breast milk. In addition to optimizing body water balance, 24-h water intake impacts fetal and maternal health. For example, acute oral fluid intake of 1–2 L increases amniotic fluid volume in women with both normal and low amniotic fluid volumes [44], but little is known about the effects of habitual maternal fluid intake on development of the child in utero or during the neonatal period. In studies involving hypotonic plasma volume expansion, plasma osmolality decreased for both mother and fetus [45], demonstrating the exchange of fluids between compartments. The change in plasma osmolality and concomitant change in plasma vasopressin has been noted in both acute water loading and 12-h fluid deprivation in pregnant women. Together with the newfound association between high vasopressin and development of preeclampsia [46], these data suggest that adequate water intake during pregnancy might be important for the health of the mother and the fetus. Given the importance of the gestational period and early life on future health outcomes, further research into maternal hydration and health is of fundamental importance. The present investigation may serve as a foundation for future research to identify practical means of monitoring hydration status and enacting behavioral change to promote adequate fluid intake during pregnancy and lactation.

In summary, the present investigation demonstrates that pregnant or lactating women, in addition to non-pregnant and non-lactating women, can use *U*_COL_ as a practical indicator of *U*_OSM_, and thus, as a reference for whether fluid intake has been adequate. Women who present with *U*_COL_ < 4 likely have a *U*_OSM_ < 500 mOsm kg^−1^, suggesting that their fluid intake is adequate to compensate for daily losses and maintain a healthy urine output. Women who present with *U*_COL_ ≥ 4 likely have a *U*_OSM_ ≥ 500 mOsm kg^−1^ and should increase fluid consumption to reduce urine concentration, thereby reducing the risk of potential health complications [19, 28–33].
